# Assessing classroom and laboratory spread of COVID-19 in a university after elimination of physical distancing

**DOI:** 10.1371/journal.pone.0283050

**Published:** 2023-03-16

**Authors:** Terri Rebmann, Travis M. Loux, Ashley Gomel, Kaeli A. Lugo, Firas Bafageeh, Haley Elkins, Lauren D. Arnold

**Affiliations:** 1 Institute for Biosecurity, College for Public Health and Social Justice, Saint Louis University, St Louis, Missouri, United States of America; 2 President’s Office, Saint Louis University, St Louis, Missouri, United States of America; 3 Department of Epidemiology and Biostatistics, College for Public Health and Social Justice, Saint Louis University, St Louis, Missouri, United States of America; 4 Saint Louis County Department of Public Health, St Louis, Missouri, United States of America; Baylor College of Medicine, UNITED STATES

## Abstract

The objective of this study was to assess COVID-19 classroom transmission in the university setting when physical distancing was eliminated. Data was collected in fall 2021 at a private university. Universal masking, robust contact tracing, vaccination requirement, and enforced testing were in place. Exposures were classified as classroom versus non-classroom. ANOVA and chi-squared tests were used to identify significant relationships between predictors and COVID-19 test result. Logistic regression was conducted to investigate the relationship between exposure type and test result. A total of 162 student cases were identified with 1,658 associated close contacts. One-third of contacts (31.1%, n = 516) only had a non-classroom exposure, 63.8% (n = 1,057) only had a classroom exposure, and 5.1% (n = 85) had both. Close contacts were significantly more likely to test positive if they had a non-classroom exposure (60 of 601; 10.0%) compared to a classroom exposure (1 of 1057; 0.1%) (OR 58.8, CI 18.5–333.3, p < 0.001). Removing physical distancing in classrooms that had universal masking did not result in high rates of COVID-19 transmission. This has policy implications because eliminating physical distancing does not greatly increase transmission risk when universal masking is in place.

## Background

The COVID-19 pandemic greatly changed the way educational institutions functioned. Many institutions of higher education (IHEs) elected to only offer online education during the 2020/2021 academic year. Those that remained open for in-person instruction had to implement extensive mitigation strategies, such as universal masking, physical distancing in classrooms and non-classroom/social spaces, reducing density on campus, diagnostic and asymptomatic surveillance testing, daily health screening for employees and students, isolation, quarantine, and contact tracing [1, 2]. These interventions were expensive and not sustainable for most IHEs [2]. Furthermore, after COVID-19 vaccine became widely available to IHE students and employees in mid-spring 2021, there appeared to be less need for such extensive public health safeguards.

Physical distancing was one of the most difficult COVID-19 mitigation strategies to implement for IHEs, because maintaining six feet of physical distance between students and faculty often reduced classroom capacity to 50% or less. Many IHEs were required to use event or meeting space as classroom space in order to accommodate physical distancing. This was not a sustainable solution, but the risk of eliminating physical distancing in classrooms was unknown. A study conducted in a K-12 school setting found that COVID-19 transmission can occur in schools when physical distancing and universal masking compliance are lacking [3], but there was not sufficient data on the impact of masking versus physical distancing.

In planning for the 2021–2022 academic year, the American College Health Association issued guidelines for reopening institutions of higher education in fall 2021 [4] that stated the need for classroom physical distancing was dependent on whether a vaccine requirement policy was implemented, which variant(s) was circulating, and community transmission rates. These ACHA guidelines were consistent with CDC guidance that indicated that some COVID-19 safeguards, including physical distancing, could be relaxed at IHEs that had high vaccine uptake in a setting of low community infection transmission rates [5]. This meant that many IHE classrooms could return to pre-pandemic capacity, with students sitting close to one another once again. However, the impact of eliminating physical distancing in IHE classrooms, even with a COVID-19 vaccine requirement policy and universal masking, was unknown.

The purpose of this study was to assess classroom spread of COVID-19 at an IHE with a COVID-19 vaccine policy that required students and employees to be up-to-date with the COVID-19 vaccination series at that time, consisting of completion of the primary vaccination series. Also in place was a universal masking requirement, but classroom physical distancing was eliminated for the fall 2021 semester. It was hypothesized that close contacts who only had masked exposure to an infected individual in a classroom would have lower infection rates compared to close contacts who had exposure(s) to an infected individual in social settings outside of the classroom.

## Methods

Data was collected for the entire fall 2021 semester (August 18—December 21, 2021) at Saint Louis University (SLU), a medium-sized Midwestern private IHE, with approximately 12,000 students and 6,000 employees who lived and/or worked on campus that semester. SLU required all employees and students to receive the COVID-19 primary vaccination series before the start of the fall 2021 semester unless they had an approved medical or religious exemption. Students and employees were required to upload proof of COVID-19 vaccination, consisting of a picture of their COVID-19 immunization card. The vaccination cards were then reviewed by a member of the SLU COVID-19 response team to determine authenticity. A universal masking policy was in place for all indoor campus spaces for the entirety of the fall 2021 semester, including both classrooms/educational spaces and non-classroom spaces such as dining areas, the student recreation center, and hallways. Employees and students were instructed to monitor and enforce the mask policy by asking the noncompliant individual to put on or wear their mask correctly. Mask noncompliance could also be reported anonymously to the SLU Office of Compliance and Ethics Team, who followed up on all reports. Mask compliance may have been enforced inconsistently. SLU COVID-19 testing protocols consisted of the following: 1) mandatory asymptomatic surveillance testing at move-in/start of the semester was required for all residential students who were less than two weeks past completing their COVID-19 primary vaccination series, 2) diagnostic testing of symptomatic individuals as needed, 3) required diagnostic testing for all close contacts identified through contact tracing who had not tested positive in the previous 90 days, 4) asymptomatic surveillance testing of student athletes and staff as per the National Collegiate Athletic Association (NCAA) guidelines [6], and 5) asymptomatic diagnostic testing of individuals identified as a potential cluster, such as all residents on a residence hall floor that experienced multiple COVID-19 cases. Diagnostic testing of symptomatic individuals consisted of using an Abbott BinaxNOW rapid antigen test followed by a single confirmatory LabCorp molecular RT-PCR collected via self-administered anterior nasal swab if the rapid antigen test was negative. Asymptomatic surveillance testing and diagnostic testing for all close contacts consisted of using a Clinical Reference Laboratory (CRL) saliva-based molecular RT-PCR. Each infected person was only counted once unless they tested positive twice more than 90 days apart. All student tests conducted in the fall semester were included for this analysis, but the only employee tests included were those collected because the employee who teaches (i.e., faculty) and was an identified close contact of an infected student in a classroom setting. Therefore, the word “faculty” will be used for the remainder of this article when reporting on employee COVID-19 tests and results; the word “employee” is used when referring to policies that affected both faculty and non-faculty employees or data summarizing all employees when it was not possible to differentiate between faculty versus non-faculty. Faculty member COVID-19 tests due to a community or household exposure were excluded from this analysis. Students and employees may have been tested more than once during the semester.

Infected students and employees were required to isolate for 10 days after the start of their symptoms or positive test result. After a case was identified, a member of SLU’s internal contact tracing team conducted a case investigation. Cases identified close contacts by name and described the type of exposure (masking status of both individuals, indoors versus outside, and in-classroom versus out-of-classroom). In addition, faculty were asked to collect seating charts for every session of their course(s) throughout the semester to aid the contact tracing team in identifying classroom close contacts while maintaining the infected individual’s anonymity.

A close contact was defined as any individual who had one or more exposures to at least one infected individual. An exposure was defined as any single encounter in which an infected individual was within six feet of another individual for 15 minutes or more within a 24-hour time period while the infected person was contagious. The period of contagiousness was considered to begin two days prior to symptom onset or a positive COVID-19 test. A close contact could have multiple exposures to the same individual or to multiple infected individuals. A *classroom close contact* was defined as a student or faculty who was within six feet of an infected individual for 15 minutes or more while in classroom setting while the infected person was contagious. If a case was unable to name the student(s) who sat within six feet of them on all sides in class, the course seating chart was requested from the faculty and used to identify the classroom close contacts. Because of the universal masking policy and assumed enforcement by employees, all classroom close contacts were imputed as a masked close contact exposure. Non-classroom close contacts were those who had an exposure to an infected individual, but were not identified as having been exposed in a classroom (i.e., the exposure took place outside of the classroom and/or in some type of social setting).

Close contacts were informed that they had been exposed to someone who tested positive for COVID-19 and were required to undergo COVID-19 molecular PCR testing five to seven days after exposure. Results of all tests, whether negative or positive, were recorded. Close contacts were required to quarantine for seven days after their exposure unless they had tested positive for COVID-19 in the past 90 days. Testing and quarantine of close contacts were monitored and enforced by the SLU Contact Tracing and Student Health Center teams. Students who were noncompliant with post-exposure testing or quarantine were referred to the Office of Student Conduct.

### Data analysis

Having *completed the primary vaccination series* was defined as having received either one dose of Janssen vaccine, the second dose of Moderna or Pfizer-BioNTech COVID-19 vaccine, or the second dose of a World Health Organization-approved COVID-19 vaccine 14 or more days before exposure. Having *received a booster dose* was defined as having received either one dose of Janssen vaccine or a dose of Moderna or Pfizer-BioNTech COVID-19 vaccine after having completed the primary vaccination series. *Receiving one dose* was defined as receipt of the second dose of Moderna or Pfizer-BioNTech 14 days or fewer days before the exposure, or receipt of one dose of Moderna or Pfizer-BioNTech COVID-19 vaccine. Those who had received no vaccine doses or a single dose of Janssen fewer than 14 days before the exposure were considered unvaccinated.

The number of exposures was calculated for each close contact. Mask status was recorded for each interaction/exposure; if either the case or close contact was unmasked, the exposure was classified as unmasked. Close contacts were also divided into those who only had a classroom exposure, had both classroom and non-classroom exposures, or had only non-classroom exposures. Other variables collected for students and faculty included sex, age, and vaccination status. Student demographics assessed included housing status (i.e., living in on- or off-campus housing), enrollment as an undergraduate or graduate student, being a student athlete, membership in a fraternity or sorority, or having clinical responsibilities through health or social work majors.

Descriptive statistics are reported as means and standard deviation for numeric variables and counts and percentages for categorical variables. ANOVA and chi-squared tests were used to identify significant relationships between demographic and other characteristics and COVID-19 test result. Logistic regression models were conducted to investigate the relationship between masked classroom exposure and positive COVID-19 test result. All statistical analyses were conducted using R software (version 4.1.1). A p-value < 0.05 was used throughout the analysis to indicate statistical significance. This project involved a retrospective study of medical records; all data were fully anonymized before analysis. The SLU Institutional Review Board approved this study (IRB # 32499) and waived the requirement for informed consent.

## Results

At the start of the fall 2021 semester, 97.1% of students and 95.8% of employees had received the COVID-19 primary vaccination series; there were 346 approved exemptions among the ~ 12,000 students and 288 approved exemptions among the ~ 6,000 employees respectively. For the fall semester (August 18—December 21, 2021), 6,022 SARS-CoV-2 tests were conducted; or these, 6,001 were student tests and 21 were faculty tests. Of the 6,001 student tests, 62.8% (n = 3,766) were diagnostic tests, and 37.2% (n = 2,235) were surveillance tests. All faculty tests were diagnostic tests consisting of asymptomatic testing after the faculty member was an identified close contact of an infected student in a classroom setting.

During the fall 2021 semester, there were two faculty cases that resulted in 11 student classroom exposures. Also that semester, 162 student cases were identified, with 1,647 associated close contacts exposed in and/or outside the classroom. No student nor faculty member tested positive more than once during the semester in which this study took place. In total, there were 1,658 associated close contacts for the semester (mean = 9.9 close contacts per case). Of all 1,658 close contacts, 72.0% (n = 1,194) were classified as being a masked exposure in which both the infected individual and close contact were masked and 28.0% (n = 464) involved exposure during which one or both individuals were unmasked (Table 1). Close contacts had an average of 1.5 exposures (range: 1–14). About one-third (31.1%, n = 516) of exposures occurred only in non-classroom settings, 63.8% (n = 1,057) took place in a classroom/educational space, and 5.1% (n = 85) of close contacts had both non-classroom and classroom exposures (Table 1). Among all 1,658 close contacts, 3.7% (n = 61) tested positive.

**Table 1:** Close contacts’ demographic characteristics, mask use, classroom exposure, and number of exposure incidents by SARS-CoV-2 test results.

**Characteristic—All**	**SARS-CoV-2 test results No. (%)**			
	**All N = 1658**	**Negative N = 1597**	**Positive N = 61**	**p value** ^a^
**Type of Exposure**				
Both classroom and non-classroom exposures	85 (5.1)	75 (4.7)	10 (16.4)	< .001
Only classroom exposure	1057 (63.8)	1056 (66.1)	1 (1.6)	
Only non-classroom exposure(s)	516 (31.1)	466 (29.2)	50 (82.0)	
**Type of Exposure—Binary**				
Any non-classroom exposure(s)	601 (36.2)	541 (33.9)	60 (98.4)	< .001
Only classroom exposure	1057 (63.8)	1056 (66.1)	1 (1.6)	
**Mask Status**				
Unmasked exposure(s)	464 (28.0)	405 (25.4)	59 (96.7)	< .001
Masked exposure(s)	1194 (72.0)	1192 (74.6)	2 (3.3)	
**Number of exposure incidents** ^b^ **(mean (SD))**	1.52 (1.06)	1.50 (1.00)	2.07 (1.99)	< .001
**Vaccination Status** ^c^				
Unvaccinated	44 (2.7)	43 (2.7)	1 (1.6)	0.23
Received one dose	18 (1.1)	16 (1.0)	2 (3.3)	
Completed primary series	1497 (90.3)	1445 (90.5)	52 (85.2)	
Received booster dose	99 (6.0)	93 (5.8)	6 (9.8)	
**Number of days after completing primary vaccination series (mean (SD))**				0.21
Unvaccinated/ incomplete	62 (3.7)	59 (3.7)	3 (4.9)	
Less than 120 days	461 (27.8)	450 (28.2)	11 (18.0)	
120 days or more	1135 (68.4)	1088 (68.1)	47 (77.0)	
**Gender**				
Male	607 (36.6)	584 (36.6)	23 (37.7)	1.00
Female	1051 (63.4)	1013 (63.4)	38 (62.3)	
**Age (mean (SD))**	21.02 (4.57)	21.03 (4.63)	20.75 (2.40)	0.45
**University Role Status**				
Student	1637 (98.7)	1576 (98.7)	61 (100.0)	0.49
Faculty member	21 (1.3)	21 (1.3)	0 (0.0)	
**Student Characteristic**	**SARS-CoV-2 test results No. (%)**			
	**All (N = 1637)**	**Negative (N = 1576)**	**Positive (N = 61)**	**p value** ^a^
**Housing Status**				
Off campus	807 (49.3)	770 (48.9)	37 (60.7)	0.09
On campus	830 (50.7)	806 (51.1)	24 (39.3)	
**Student Level**				
Undergraduate	1409 (86.1)	1356 (86.0)	53 (86.9)	1.00
Graduate	228 (13.9)	220 (14.0)	8 (13.1)	
**Student Athlete**				
No	1506 (92.0)	1457 (92.4)	49 (80.3)	= .001
Yes	131 (8.0)	119 (7.6)	12 (19.7)	
**Attended an Identified Social Gathering** ^d^				
No	1196 (73.1)	1183 (75.1)	13 (21.3)	< .001
Yes	441 (26.9)	393 (24.9)	48 (78.7)	
**Student in a Greek Organization**				
No	1376 (84.1)	1326 (84.1)	50 (82.0)	0.78
Yes	261 (15.9)	250 (15.9)	11 (18.0)	
**Student Had Clinical Responsibilities**				
No	1517 (92.7)	1464 (92.9)	53 (86.9)	0.13
Yes	120 (7.3)	112 (7.1)	8 (13.1)	

^a^ Determined by Chi-square test for all comparisons except for number of exposure incidents, number of days after completing primary vaccination series, and age (continuous variables), which were assessed using a *t* test.

^b^ Exposure incident = an encounter in which a susceptible individual was within 6 feet of someone with COVID-19 for ≥ 15 minutes during a single 24-hour time period.

^c^Unvaccinated = Received no vaccine doses or one dose ≤ 2 weeks before the exposure. Received one dose = ≥ 2 weeks after receiving one dose of Moderna or Pfizer-BioNTech COVID-19 vaccine, but has not received the second dose before the exposure. Completed primary series = ≥ 2 weeks after receiving second dose of Moderna or Pfizer-BioNTech COVID-19 vaccine, but has not received a booster dose(s) before the exposure. Received booster dose = Received either one dose of Janssen vaccine or a dose of Moderna or Pfizer-BioNTech COVID-19 vaccine after having completed the primary vaccination series before the exposure.

^d^Identified social gathering = a group of 10 or more individuals who spent time together in a social, non-classroom setting while at least one attendee was contagious.

Close contacts were significantly more likely to test positive if they had an exposure in a non-classroom setting (60 of 601; 10.0%) compared to those who only had a classroom exposure (1 of 1057; 0.1%) (OR 117.1, CI 25.8–2072.6, p < 0.001; Table 1). In additional univariate analyses, those who had a higher number of exposure incidents were also more likely to test positive (p < 0.001). After adjustment for this characteristic, the relationship between only classroom exposure and positive test remained (aOR 110.4, CI 24.2–1955.5, p < 0.001).

For the fall semester (August 18—December 21, 2021), there were 223 student COVID-19 cases total: 162 student cases who were not identified as close contacts and 61 were close contacts who tested positive on their post-exposure test. Of the 2,235 surveillance tests conducted, 5 were positive (0.2% positivity rate); in contrast, 5.8% of the 3,755 diagnostic tests were positive (n = 218). Of the 3,755 diagnostic tests conducted, 2,361 (62.9%) involved a symptomatic individual. The overall campus positivity rate for the semester was 3.7% (223 cases out of 6,001 tests conducted). Cluster testing accounted for 58 of the 3,766 diagnostic tests conducted, of which 10 were positive (positivity rate among cluster testing participants: 17.2%).

## Discussion

This study found that removing physical distancing in IHE classrooms that previously maintained universal masking did not result in high rates of COVID-19 transmission. Infection rates were significantly higher among individuals who had close contact outside the classroom or a combination of classroom and non-classroom exposures versus among those who only had a classroom exposure. It is very likely that this is due to the use of universal masking in classrooms during that semester, because all of those who reported only having a classroom exposure had a masked exposure versus while those who had a non-classroom exposure with or without a classroom exposure had at least some unmasked exposure to an infected individual. This provides evidence that mask use remains critical in preventing classroom transmission of SARS-CoV-2 when physical distancing is eliminated. It also reinforces past research that found that most IHE campus outbreaks have been related to unmasked gatherings outside the classroom, congregate living arrangements, university athletics, and/or school breaks rather than transmission in classrooms or laboratory spaces [3, 7, 8].

The findings of this study are important because previous research has not assessed the impact of physical distancing in classrooms separate from the protection provided by universal masking. Although it is not possible to state definitively that physical distancing can safely be eliminated from educational spaces without increasing the risk of COVID-19 transmission, this study found that positivity rates were very low when physical distancing was eliminated while mask use was retained. It may also provide some level of comfort for IHE faculty who are concerned about classroom exposure [9]. Should situations arise where classroom masking requirements may be implemented by an IHE, such as during a future COVID-19 surge or outbreak of influenza, this study also provides additional evidence to support a modified contact tracing process, one that would not need to include determining classroom close contacts. This is important because during larger outbreaks, contact tracing can require substantial human resources and time to locate contacts and follow them for testing outcomes. The ability to eliminate the need for identifying classroom contacts will help contact tracing teams focus their often-limited resources on identifying and tracking those contacts at highest risk.

This study took place at a university that implemented a COVID-19 vaccination requirement for the 2021/2022 academic year. Due to this policy, vaccination rates were very high: 97.1% for students and 95.8% for employees. Because of this high compliance, inclusion of vaccination status as a predictor of infection was not possible. While the high vaccination rate likely contributed to the overall low impact of COVID-19 on campus that semester, despite the high vaccine breakthrough associated with the Delta variant [10], masking helped reduce transmission despite the lack of physical distancing in classroom spaces.

In the semester after this study was conducted, COVID-19 response shifted from focusing on preventing all disease transmission to instead aiming to limit severe disease and negative impacts on healthcare systems [11]. This change involved shortening isolation and quarantine periods, elimination of physical distancing in most settings, recommended universal masking only when community risk levels are high, and emphasis on focusing contact tracing in high-risk settings, such as hospitals or nursing homes. Despite this, the findings from this study provide valuable evidence about the lack of need for physical distancing for responding to this pandemic, future outbreaks of COVID-19 once the disease fully shifts to being endemic, and other future outbreaks and pandemics from influenza and other emerging infectious diseases. This is critical evidence because implementation of physical distancing in IHE classrooms and lab settings was very challenging, expensive, and not sustainable.

Some limitations of this study must be noted. Information gathered regarding where and when exposures took place may not have been completely accurate due to social desirability bias (if infected students did not want to identify their friends as close contacts) or poor historical memory, which could have affected the findings. Although seating charts were obtained for each class after a classroom exposure was identified, it is possible that the students and faculty did not remain in that exact configuration for the duration of class (e.g. rearrangement for small group activities), which could have resulted in missing individuals with a classroom exposure. It is also possible that additional unrecognized exposures occurred involving asymptomatic individuals that could have affected these findings. This study took place in the fall 2021 academic semester when the SARS CoV-2 Delta variant was circulating. Although the Delta variant was found to be more transmissible than the ancestral strain of SARS CoV-2, it was less transmissible than the Omicron variant which began circulating in late 2021 and most of 2022. It is unknown if this would have had an impact on COVID-19 transmission in classrooms.

## Conclusion

Proposals to eliminate physical distancing in classrooms and laboratories of institutes of higher education mid-pandemic raised concerns about classroom-based transmission. However, these changes resulted in almost no increase in cases in this study. The use of universal masking, a COVID-19 vaccination requirement policy, robust contact tracing, isolation and quarantine, and other COVID-19 mitigation strategies aided in minimizing campus transmission in educational spaces once physical distancing was removed. Elimination of physical distancing in classrooms and lab spaces allows institutes of higher education to return to pre-pandemic space configurations and may have implications for how to manage future surges of COVID-19 or other similar respiratory illness on campus. This in turn enhances student learning and faculty satisfaction while not contributing to campus disease spread.
